# Assessments of serum copper and zinc concentration, and the Cu/Zn ratio determination in patients with multidrug resistant pulmonary tuberculosis (MDR-TB) in Côte d’Ivoire

**DOI:** 10.1186/s12879-017-2343-7

**Published:** 2017-04-11

**Authors:** Gnogbo Alexis Bahi, Lydie Boyvin, Souleymane Méité, Gervais Melaine M’Boh, Kadjowely Yeo, Kouassi Raymond N’Guessan, Alain Dit Philippe Bidié, Allico Joseph Djaman

**Affiliations:** 1 Department of clinical and Fondamental Biochemistry, Pasteur Institute of Cote d’Ivoire (IPCI), 01 BP 490, Abidjan, 01 Côte d’Ivoire; 2 Pharmacodynamics Laboratory of Biochemical, University FélixHouphouët- Boigny (UFHB), Abidjan 01 BP V34, Abidjan, 01 Côte d’Ivoire

**Keywords:** Abidjan, Trace elements, Cu/Zn Ratio, MDR-TB, Second-line anti-TB drugs

## Abstract

**Background:**

In Côte d’Ivoire, multidrug-resistant tuberculosis (MDR-TB) is a serious public health problem with a prevalence estimated at 2.5% in 2006. Zinc and copper are essential Trace element needed to strengthen the immune system and also useful in the fight against tuberculosis. The Cu / Zn ratio is a good indicator of oxidative stress.

The principal aim of this study was to evaluate the serum concentration of some trace element and determine the Cu / Zn ratio in patients with multidrug resistant pulmonary tuberculosis (MDR-TB) before and after second line treatment of TB.

**Methods:**

Blood samples were obtained from 100 MDR-TB patients after confirmation of their statu*s* through the microscopic and molecular diagnosis of resistance to Isoniazid and Rifampicin by GeneXpert. The concentration level of zinc and copper were determined using flame air / acetylene atomic absorption spectrometer (AAS) Type Varian Spectr AA-20 Victoria, Australlia.

**Results:**

A significant decrease in zinc levels (*P* < 0.05) and an increased Cu / Zn ratio (*P* < 0.05) was observed in MDR-TB patients compared to controls TB free. During treatment a significant reduction in Cu / Zn ratio (*P* < 0.05) was observed compared to the initial result.

**Conclusions:**

The decrease in serum zinc level and the high Cu / Zn ratio could explain the immune system dysfunction and the high level of oxidative stress in patients with MDR-TB. Therefore the evaluation of the zinc and copper status could represent essential parameters in monitoring of TB second line treatment for better treatment management.

**Electronic supplementary material:**

The online version of this article (doi:10.1186/s12879-017-2343-7) contains supplementary material, which is available to authorized users.

## Background

The fight against multidrug-resistant TB (MDR-TB) is a big challenge. In 2014, the World Health Organization (WHO) estimated 480,000 the number of people who contracted MDR-TB and about 190,000 deaths attributed to this form of TB [1]. The WHO report, showed an overall net increase of cases of MDR-TB from 42% in 2012, 3.6% of new TB cases and 20.6% of previously treated cases are MDR-TB [1]. According to the report, less than 50% of patients with MDR-TB detected in 2010 were successfully treated. These figures reflect the high mortality rate associated with this form of TB.

In Côte d’Ivoire, the national tuberculosis control program (NTBC) reported that the recent socio-political crisis in Côte d’Ivoire has encouraged the emergence of the multidrug resistance pulmonary TB form of which prevalence is estimated to 2.5% in 2006 [2]. The outbreak of this disease and its spread are due to malnutrition and deterioration of the immune system [3, 4]. Other studies have reported that some TB drugs could affect the nutritional profile of the TB patient receiving treatment [5, 6].

The existence of a close relationship between micronutrients and immune system modification has been reported in many studies, in Indonesia [7] and Ethiopia [8]. Among these micronutrients, zinc and copper play an important role in the proliferation and differentiation of cells of innate and acquired immunity [9]. Moreover, the Cu / Zn ratio is an indicator for assessing the level of oxidative stress in the case of infectious diseases in general [10, 11]. These two micronutrients play an essential role in resistance to certain infectious diseases like tuberculosis [12]. However, little study has been carried out on the micronutrient status of patients with MDR-TB.

The aim of our study is to evaluate serum copper and zinc level and determine the Cu / Zn ratio in MDR-TB patients before treatment, after 3 months treatment and then after 6 months second line TB treatment, respectively.

## Methods

### Study population and environment

This is a prospective and experimental study that was conducted from January 2014 to December 2015 at the Institut Pasteur of Côte d’Ivoire (IPCI). Microscopy and molecular analyzes were performed at the national laboratory for tuberculosis chosen patients from the five antituberculosis centers (ATC) of Abidjan. It involved blood samples from 100 MDR-TB patients (50 women and 50 men aged between 18 and 55 years).

The blood samples used in our study were from MDR-TB patients who received second-line treatment after failure of first-line treatment with positive microscopy and after Gene Xpert [13] confirmation of resistance to at least two TB drugs (Isoniazid and Rifampicin).

### Sampling and blood collection

The sampling was carried out at various stages of the patient’s treatment follow up as outlined below:

M0 stage (100 samples) the initial stage after confirmation of the multidrug resistance test as mentioned above and before any treatment commences.

M3 stage (100 samples) and M6 stage (100 samples) were followed up stages after 3 and 6 months of second-line anti-TB drugs treatment respectively.

A total of 300 blood samples from 100 MDR-TB patients and 100 samples from volunteers’ non-tuberculosis to serve as control, living in the same environment age between 18 and 55 years were selected for this study.

Fasting blood was collected in anticoagulant-free tubes (red top tubes). The samples were then centrifuged at 3000 rounds / minute for 5 min using a centrifuge horizon 642 VES THE DRUCKER CO., USA and the serum collected in Ependorf® tubes were stored at −20 °C.

### Serum zinc and copper measurement

The serum copper and zinc measurement was performed using an air / acetylene flame atomic absorption spectrometer (AAS) Varian model Spectr AA - 20 Victoria, Australlia [14].

The blood tubes used were immersed in a nitric acid solution (HNO_3_) at 10% (*v*/v), following a previous day (12 h) washing in a solution of hydrochloric acid (HCl) at 10% (*v*/v) . They were then rinsed twice with distilled water and dried. Protein precipitation was made by diluting 1 mL of serum in 4 mL of a solution of hydrochloric acid (2 M). After homogenization according to the method of Banjoko et al. [15], each sample was allowed to settle. The clear supernatant obtained was sucked directly into the flame atomic absorption spectrophotometer at the wavelength of 324.8 nm; 213.9 nm for the copper and zinc, respectively. A multi element-standard solution 1000 ppm (Merck, USA) previously diluted at 1/500 with nitric acid-deionized water (0.03 M) was used to prepare the calibration range (0; 0.5; 1.5; 2.0; 4 ppm). The concentration measurements were performed in triplicate and adjusted against the white (HCl solution 2 M).

The normal reference values for serum copper, zinc were respectively 11.0–22.0 μmol/L, 11.5–18.5 μmol/L [9].

### Statistical analysis

The mean values followed by the standard error of the mean (mean ± SEM) of data were performed using the Graph Pad Prism 5.0 software (Microsoft, USA). Statistical analysis of results was performed using analysis of variance (ANOVA) followed by a multiple comparison Tukey test. The difference is significant when the *p*-value < 0.05.

## Results

The results obtained from the blood assessment showed a reduced serum Zn concentration level at the initial stage of TB treatment compared to control, (*P* < 0.05) in men (3.049 ± 0.35 μmol/L) and in women (3.866 ± 0.45 μmol/L). After 3 and 6 months of treatment (M3 & M6), it was noted a significant increase in the serum Zn level (*P* < 0.05) compared to M0 stage. The values of zinc obtained during treatment were: men M3 (6.35 ± 0.50 μmol/L), M6 (6.99 ± 0.37 μmol/L); M3 women (5.61 ± 0.90 μmol/L), M6 (5.95 ± 0.52 μmol/L) respectively.

**Fig. 1:**
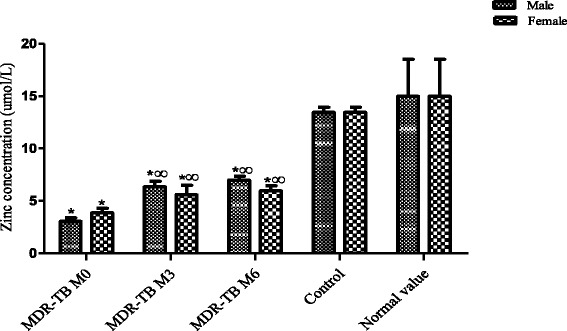
Zinc concentration in MDR-TB and control. *: Significant difference between MDR-TB and the control population. ^∞:^ Significant difference between the stage M0 and M3 § M6 stages

All the same the Zn concentration level still remained inferiors to the normal values and to those of control population (Figure 1).

**Fig. 2:**
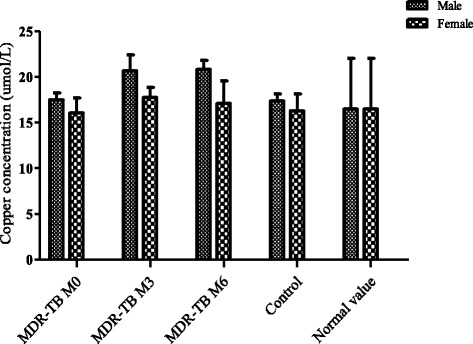
Copper concentration in MDR-TB and control

The serum copper concentrations were normal before and after 6 months of treatment, in both sexes, compared to controls (Figure 2).

**Fig. 3:**
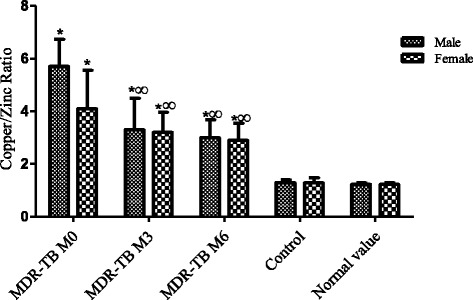
The Cu / Zn Ratio in MDR-TB and control. *: Significant difference between MDR-TB and the control population. ^∞:^ Significant difference between the stage M0 and M3 § M6 stages. MDR-TB: Multi drug resistant tuberculosis . M0: Before treatment. M3 and M6: Three and six Months after treatment

However, the Cu / Zn ratio, which was very high at the initial stage (M0) dropped significantly (*P* < 0.05) during TB second-line treatment (M3 & M6). Nevertheless, compared to non-tuberculosis control population the ratio value remained high (*P* < 0.05) (Table 1 and Figure 3).

**Table 1:** Values of the Cu / Zn ratio in patients with MDR-TB at different monitoring stages

MDR-TB Patients	Cu / Zn ratio during the treatment follow up stages			Control	Normal Value
	M0	M3	M6		
Male	5.71 ± 2.0^a^	3.34 ± 3.2^ab^	3.08 ± 0.7^ab^	1.29 ± 0.1	1.14–1.29
Female	4.13 ± 2.5^a^	3.26 ± 0.7^ab^	2.93 ± 0.6^ab^	1.21 ± 0.1

## Discussion

We noted in this study, a significant decrease in zinc concentrations in MDR-TB patients compared to the non-tuberculosis control population (*P* < 0.05). These zinc concentration values remained lower despite a significant increase in value (*P* < 0.05) during TB treatment comparable to the initial stage before treatment. These results are consistent with those of Edem et al. [6, 16] who reported, reduced zinc concentration during tuberculosis with an increase in these concentrations after 4 and 6 months of treatment respectively. These authors indicated that the lower zinc concentration observed compared to normal values was due to a redistribution of zinc flowing in other tissues including the liver tissue; this could be explained due to a reduced liver’s production of α-2-macroglobulin (a protein carrier of zinc in the blood) for the benefit of high production of metallothionein, a protein carrying zinc to the liver [6]. Zinc insufficiencies among MDR-TB patients have a negative impact on the immune system. In effect, zinc deficiency is responsible for an alteration in the macrophage function and a reduction of production of tumor necrosis factor (TNF-α) and interferon-γ (INF-γ). These lower zinc concentrations would also be responsible for the decrease in the proliferation and differentiation of T and B lymphocytes [17, 18]. The immunity factors would be at the forefront in the performance of immune system defenses and protect against active tuberculosis [19]. In addition, it was reported that zinc and vitamin A supplementation in adult patients with active TB would allow elimination of *Mycobacterium*, thus leading to quick cure of these patients [7]. The zinc deficiency could thus explain the longer second-line TB treatment period among MDR-TB. While normal copper concentration values were observed in MDR-TB with or without treatment compared to the control population and normal values. These normal copper concentrations could be explained by the non-specificity of the synthesis of copper transport proteins including ceruloplasmin [8]. However, a very high Cu / Zn ratio was observed in MDR-TB compared to non-tuberculosis control. However, during treatment, the Cu / Zn ratio experienced a significant reduction compared to the initial assessment; but comparing to non-tuberculosis control population, it has increased significantly. High values of the ratio Cu / Zn in patients with active TB were reported by the work of Ceftci et al. [20]. These high values of the Cu / Zn ratio were due to the drop in the zinc concentrations; that means a high level of oxidative stress in these MDR-TB patients and negatively affect the immune system [21]. Moreover, the Cu/Zn ratio is an indicator of the nutritional status of zinc in patients. A Cu / Zn ratio greater than 2 means severe bacterial infection.

## Conclusion

This study highlighted a significant reduction in serum zinc concentrations among multidrug-resistant tuberculosis in **C**ôte d’Ivoire. This deficiency in zinc with a Cu / Zn ratio values well above normal value is responsible for the dysfunction in the immune system and raising the level of oxidative stress. The persistence deficiency in zinc despite 6 months of second-line TB treatment require zinc supplement intake in these MDR-TB in addition to anti-tuberculosis molecules. This supplementation would boost the immune system and restore the balance between the production of free radicals and antioxidants in this MDR-TB. This will enhance the effectiveness of the TB treatment and prevent new resistance to second-line TB drugs.

## Additional file

Additional file 1: Analysis results. Results of copper, zinc and the Zn / Cu ratio of MDR-TB and controls with enrollment centers (ATC), sex, and follow-up stage for TBMR. (XLSX 30 kb)

## Abbreviations

AAS: Atomic absorption spectrometer
ATC: Anti-tuberculosis centers
Cu: Copper
INF-γ: Interferon-γ
IPCI: Institute Pasteur of Côte d’Ivoire
MDR-TB: Multidrug﻿ ﻿﻿resistant tuberculosis
TB: Tuberculosis
TNF-α: Tumor necrosis factor alpha
Zn: Zinc
